# Kompetenzorientiertes Prüfen: die strukturierte mündliche Prüfung

**DOI:** 10.1007/s00106-025-01687-0

**Published:** 2025-11-26

**Authors:** Susanne Isabella Günther, Theresa Lüdke, Marie-Luise Polk, Thomas Zahnert, Marcus Neudert

**Affiliations:** Klinik und Poliklinik für Hals‑, Nasen-, und Ohrenheilkunde, Universitätsklinikum Carl Gustav Carus an der TU Dresden, Fetscherstr. 74, 01307 Dresden, Deutschland

**Keywords:** Constructive Alignment, Medizinische Ausbildung, Prüfungsbeurteilung, Standardisierung, Klinische Entscheidungsfindung, Constructive alignment, Medical education, Educational measurement, Standardization, Clinical reasoning

## Abstract

**Hintergrund:**

Strukturierte mündliche Prüfungen (SMP) sind ein valides Instrument zur Abbildung von Handlungs- und Begründungswissen (Stufe 2). Sie schließen die Lücke zwischen schriftlichen Formaten (Faktenwissen, Stufe 1) und praktischen Formaten wie „objective structured clinical evaluation“ (OSCE; Handlungskompetenz, ab Stufe 3). Durch standardisierte Fallvignetten, Checklisten und definierte Bewertungsmaßstäbe bieten SMP Potenzial für Objektivität, Reliabilität und Vergleichbarkeit.

**Ziel:**

Am Beispiel der Reintonaudiometrie (RTA) und Rhinomanometrie (RMM) wurden Durchführbarkeit, Bewertungsgüte (interne Konsistenz, Trennschärfen, Faktorenanalyse) und Prüfer:inneneffekte einer SMP gemäß Kompetenzstufe 2 des Nationalen Kompetenzbasierten Lernzielkatalogs Medizin (NKLM) untersucht.

**Material und Methoden:**

Im curricularen Unterricht wurden RTA (*n* = 217) und RMM (*n* = 190) vermittelt und anschließend in einer SMP geprüft. Bewertet wurden 5 Kategorien (Benennung, Durchführung, Beschreibung, Interpretation, Differenzialdiagnose; maximal 27 Punkte) mit Abzügen bei Hilfestellungen. Acht ärztliche Prüfer:innen führten die Prüfungen im 1:1-Setting durch. Neben deskriptiver Statistik erfolgte eine einfaktorielle ANOVA („analysis of variance“) zur Analyse von Prüferunterschieden.

**Ergebnisse:**

Die mittlere Gesamtpunktzahl betrug 25,20 ± 1,94 (RTA) bzw. 24,84 ± 1,94 (RMM). Höchste Werte zeigten Benennung und Durchführung, niedrigste Werte fanden sich für Beschreibung (RTA) bzw. Interpretation (RMM). Für die RTA ergaben sich signifikante Prüferunterschiede (*p* = 0,001), für die RMM keine (*p* = 0,078).

**Schlussfolgerung:**

Die SMP erwies sich als praktikables und reliables Format zur Abbildung von Handlungs- und Begründungswissen. Der Nachweis einzelner Prüferunterschiede verdeutlicht die Notwendigkeit von Prüfer:innenschulungen, Kalibrierung und standardisierten Instrumenten. Damit kann die SMP wesentlich zur Umsetzung kompetenzorientierter Prüfungen im Medizinstudium beitragen.

## Abstract (en)

**Background:**

Structured oral examinations (SOEs) are valid instruments to assess applied knowledge and reasoning skills (competency level 2). They bridge the gap between written examinations focusing on factual knowledge (level 1) and practical formats such as objective structured clinical evaluations (OSCEs) assessing performance skills (from level 3). Through standardized case vignettes, checklists, and defined scoring criteria, SOEs offer potential for enhanced objectivity, reliability, and comparability.

**Objective:**

Using pure tone audiometry (PTA) and rhinomanometry (RMM) as examples, this study examined the feasibility, psychometric quality (internal consistency, item discrimination, factor analysis), and examiner effects of an SOE designed according to the *Nationaler Kompetenzbasierter Lernzielkatalog Medizin* (NKLM), competency level 2.

**Methods:**

Within the curricular ENT teaching program, PTA (*n* = 217) and RMM (*n* = 190) were taught and subsequently assessed in an SOE. Five categories were rated (identification, performance, description, interpretation, and differential diagnosis; maximum 27 points), with point deductions for examiner assistance. Eight medical examiners conducted the exams in a 1:1 setting. Descriptive statistics and a one-way analysis of variance (ANOVA) were used to analyze examiner differences.

**Results:**

The mean total score was 25.20 ± 1.94 for PTA and 24.84 ± 1.94 for RMM. The highest scores were obtained for identification and performance, the lowest for description (PTA) and interpretation (RMM). Significant examiner differences were found for PTA (*p* = 0.001) but not for RMM (*p* = 0.078).

**Conclusion:**

The SOE proved to be a feasible and reliable format for assessing applied knowledge and reasoning. The observed examiner differences highlight the need for examiner training, calibration, and standardized scoring instruments. Structured oral examinations can substantially contribute to implementing competency-based assessment in medical education.

## Wandel ärztlicher Ausbildung

Die ärztliche Ausbildung in Deutschland befindet sich in einem tiefgreifenden Wandel hin zu einer stärkeren Kompetenzorientierung nach internationalen Modellen wie dem kanadischen Referenzmodell CanMEDS-Framework.

Der Nationale Kompetenzbasierte Lernzielkatalog Medizin (NKLM 2.0) beschreibt Lernziele in 4 aufeinander aufbauenden Kompetenztiefen und dient zunehmend als Referenzrahmen für die curriculare Entwicklung [1–3]. Damit rückt Handlungs- und Begründungswissen in den Fokus. Prüfungen übernehmen eine Schlüsselrolle, da sie das Lernverhalten maßgeblich steuern und im Sinne der konstruktiven Anpassung („constructive alignment“) mit Lehrzielen und -methoden verzahnt sein müssen [4–6].

## Stellenwert mündlicher Prüfungen

Mündliche Prüfungen haben traditionell einen hohen Stellenwert, da sie komplexes klinisches Denken, Entscheidungsprozesse und kommunikative Kompetenzen in Echtzeit sichtbar machen. Klassische unstrukturierte Kolloquien weisen jedoch deutliche Schwächen auf: geringe Reliabilität, systematische Bewertungsfehler (z. B. Halo‑, Kontrast- oder Zentraltendenzeffekte) sowie eine eingeschränkte Inhaltsvalidität, wenn vorwiegend Faktenwissen geprüft wird. Diese Defizite sind seit Langem empirisch belegt [7–10].

## Konzept und Evidenz der strukturierten mündlichen Prüfungen

Zur Überwindung der bekannten Schwächen unstrukturierter Kolloquien wurde international die „structured/standardised oral examination“ (SOE)/strukturierte mündliche Prüfung (SMP) entwickelt. Kennzeichnend sind u. a. Blueprinting, standardisierte Fallvignetten mit Leitfragen, checklistenbasierte Bewertung, mehrere unabhängige Prüfer:innen sowie gezielte Prüfer:innenschulungen. Die zentralen Elemente der SMP und ihr jeweiliger Beitrag zur Testgüte sind in Tab. 1 zusammengefasst.

**Tab. 1:** Elemente der strukturierten mündlichen Prüfung (SMP) mit jeweiliger Funktion und Beitrag zur Testgüte.

Element	Funktion	Beitrag zur Testgüte
Blueprint (Prüfungsmatrix)	Definiert Themen, Gewichtung, kognitive Ebene	↑ Inhalts- und Konstruktvalidität
Fallvignetten mit Leitfragen	Standardisieren Stimulus und Antwortraum	↑ Objektivität, ↑ Vergleichbarkeit
Check- und Bewertungslisten	Legen Schlüsselinhalte und Punktvergabe fest	↑ Reliabilität, ↑ Justiziabilität
Mehrere Prüfer:innen	Parallelbewertung oder Konsens	↑ Inter-Rater-Reliabilität
Prüfer:innenschulung	Sensibilisierung für Bewertungsfehler	↓ Systematische Verzerrungen

Metaanalysen zeigen, dass SMP-Formate bei adäquatem Design Reliabilitätskoeffizienten > 0,8 erreichen und die Validität klassischer Kolloquien deutlich übertreffen [11, 12]. Dem stehen ein erhöhter organisatorischer Aufwand (z. B. Entwicklung eines Item-Pools, Prüferbriefings, Logistik) und Zeitrestriktionen gegenüber. Für die Praxis verdeutlicht die Übersicht in Tab. 1, dass bereits einzelne strukturierende Elemente, wie z. B. Fallvignetten mit Leitfragen oder prüferunabhängige Checklisten, die Objektivität und Vergleichbarkeit mündlicher Prüfungen deutlich verbessern können.

## Anwendung mündlicher Prüfungen in Deutschland

- In den Staatsexamina (M1, M3) gelten mündliche Prüfungen als letzter „unstrukturiert gebliebener“ Teil des Prüfungsportfolios. Zwar finden Absprachen zwischen Fächern statt, doch ein einheitlicher Blueprint ist selten; Erwartungshorizonte bleiben meist implizit.
- Die Facharztprüfungen werden in der Literatur als „blackbox“ kritisiert. Glaab et al. [13] und Eichhorn et al. [14] fordern die Einführung standardisierter Guided-Question-Pools, wie sie in europäischen Board-Examinations (EBEORL-HNS) erfolgreich eingesetzt werden.
- Erste Pilotprojekte an deutschen Fakultäten zeigen, dass selbst eine partielle Strukturierung (z. B. 4‑Fragen-Raster pro Fall) die Reliabilität erhöht, ohne den individuellen Prüferstil vollständig einzuschränken.

Darüber hinaus richten Fakultäten ihre Curricula zunehmend an den Lernzielen des NKLM aus und prüfen, ob Lehrinhalte die geforderten Kompetenztiefen erreichen. Albrecht et al. [1] betonen in diesem Zusammenhang den Vorteil des Kompetenzmodells, das einen kontinuierlichen und möglichst reibungslosen Übergang vom Studium in die ärztliche Weiterbildung erleichtert.

## Zielsetzung der Studie

Trotz der theoretisch gut belegten Vorteile fehlen im deutschsprachigen Raum belastbare empirische Daten zur Umsetzung und Bewertungsgüte der SMP. Diese Studie untersucht exemplarisch 2 häufig gelehrte HNO-Untersuchungsmethoden, die Reintonaudiometrie (RTA, Ohr) und die Rhinomanometrie (RMM, Nase), deren NKLM-Lernziele explizit auf Kompetenztiefe 2 (Handlungs- und Begründungswissen) ausgerichtet sind. Die Fokussierung auf je ein Verfahren aus einem unterschiedlichen Bereich dient dazu, die Übertragbarkeit der SMP-Struktur auf inhaltlich unterschiedliche Kontexte zu verdeutlichen.

Konkret wird geprüft,

- ob sich eine SMP mit Fallvignetten, Leitfragen und checklistenbasierter Bewertung entwickeln und innerhalb eines realistischen Zeitrahmens durchführen lässt (Durchführbarkeit),
- inwiefern die Bewertungsstruktur psychometrisch geeignet ist (interne Konsistenz, Trennschärfen; explorative Faktorenanalyse zur Konstruktvalidität), und
- ob sich Prüfer:inneneffekte in den vergebenen Gesamtpunktzahlen nachweisen lassen (Differenzen im Bewertungsniveau zwischen Prüfenden).

## Methode

Die Entwicklung der SMP erfolgte auf Grundlage der curricular definierten Lernziele des NKLM (Kompetenzstufe 2). Die Auswahl der Untersuchungsmethoden, die Formulierung der Leitfragen sowie die Gewichtung der Bewertungskategorien wurden im Rahmen strukturierter Interviews mit Expert:innen aus der HNO und Medizindidaktik (*n* = 5) abgestimmt.

Die Auswahl der Themenbereiche RTA und RMM erfolgte aus einem Gesamtpool von 6 im Unterricht vermittelten und für die SMP entwickelten Untersuchungseinheiten (RTA, RMM, Video-Kopfimpulstest, kalorische Prüfung, Olfaktometrie und Prick-Test). Im Rahmen der Prüfung erhielt jede:r Studierende zufällig 2 unterschiedliche Befunde aus diesem Pool, zu denen die SMP durchgeführt wurde.

Die Fokussierung auf RTA und RMM in der vorliegenden Arbeit dient der exemplarischen Darstellung des Prüfungsformats und seiner psychometrischen Evaluation, nicht der fachspezifischen Bewertung dieser beiden Verfahren. Beide Themen sind im NKLM curricular verankert: Die RTA entspricht den Lernzielen VII.2-10.1.1 („grundlegende apparative Untersuchungsmethoden für das Sinnesorgan Ohr“) und VII.2-10.2.1 („erweiterte apparative Methoden für das Sinnesorgan Ohr“), während die RMM dem Lernzielen VII.2-10.2.3 („erweiterte apparative Methoden für das Sinnesorgan Nase“) zugeordnet ist.

Diese Lernziele verlangen die Fähigkeit, Indikationen zu stellen, Kontraindikationen und Komplikationen zu benennen, Untersuchungsverfahren korrekt durchzuführen, Befunde zu interpretieren und daraus weitere diagnostische und therapeutische Maßnahmen abzuleiten. Damit bilden RTA und RMM exemplarisch die intendierte Kompetenzebene des Handlungs- und Begründungswissens gemäß NKLM-Kompetenzstufe 2 ab und sind inhaltlich geeignet, die Funktionsweise und Eignung der SMP für anwendungsorientierte Prüfungen im Medizinstudium zu demonstrieren.

Im lehrplanmäßig verankerten Unterricht in der Hals-Nasen-Ohren-Heilkunde wurden die Untersuchungsverfahren RTA und RMM jeweils in zwei 45-minütigen Unterrichtseinheiten (2 UE) sowohl theoretisch als auch praktisch vermittelt. Ziel war es, die zugrunde liegenden Lernziele gemäß NKLM auf Kompetenztiefe 2 (Handlungs- und Begründungswissen) abzubilden. Die Studierenden befanden sich zum Zeitpunkt der Lehreinheiten im Rahmen des Blockpraktikums im 10. Fachsemester des Studiengangs Humanmedizin. Jede:r Studierende absolvierte am Ende des ein-wöchigen Blockpraktikums eine strukturierte mündliche Prüfung aus 2 zufällig ausgewählten Befunden aus einem vordefinierten Pool (RTA, RMM, kalorische Prüfung, Video-Kopfimpulstest, Olfaktometrie oder Prick-Test). In die vorliegende Auswertung gingen ausschließlich Prüfungen ein, in denen mindestens eine der beiden Diagnostiken RTA oder RMM geprüft wurde. Dabei ergaben sich in der Kohorte des Jahres 2023 *n* = 24 Studierende, die sowohl in Bezug auf RTA als auch RMM geprüft wurden, und *n* = 74 RMM bzw. *n* = 77 RTA mit jeweils einem anderen Befund. In der Kohorte 2024 waren es *n* = 47 Studierende, die hinsichtlich der RTA und RMM geprüft wurden, sowie jeweils *n* = 45 RMM bzw. *n* = 69 RTA. Das Geschlecht entspricht in etwa der typischen Geschlechtsverteilung im Medizinstudium in Deutschland mit 70 % (Kohorte 2023)/71 % (Kohorte 2024) weiblichen und 30 %/29 % männlichen Studierenden [15].

Bewertungsgrundlage bildete eine checklistenbasierte Prüfungsstruktur mit 5 inhaltlichen Kategorien:

1. *Benennung der Diagnostik* (1 Punkt): korrekte Nennung des Namens der jeweiligen Untersuchung.
  *„Wie heißt diese Untersuchung?“*
2. *Durchführung* (6 Punkte): schrittweise Beschreibung der Untersuchung einschließlich vorbereitender Maßnahmen und Materialeinsatz.
  *„Wie wird diese Diagnostik durchgeführt?“*
3. *Beschreibung* (6 Punkte): korrekte Benennung der diagnostischen Parameter (z. B. Achsenbeschriftung, Farbkodierung, Kurvenform).
  *„Was ist auf der Diagnostik dargestellt“*
4. *Interpretation* (6 Punkte): Befundbewertung im klinischen Kontext (z. B. Schallleitungs- vs. Schallempfindungsschwerhörigkeit bzw. knöcherne vs. funktionelle Nasenwegseinengung).
  *„Werten Sie die vorliegende Diagnostik aus!“*
5. *Differenzialdiagnostik* (4 Punkte): Nennung von 2 plausiblen Erkrankungen.
  *„Welche Erkrankung könnte vorliegen?“*

Für die Kategorien 2–5 wurde zusätzlich erfasst, ob Hilfestellung durch die prüfende Person erforderlich war. Jede aktive Hilfestellung durch die Prüfenden führte zu einem Abzug von 1 Punkt (maximal 4 Punkte Abzug).

Die Checkliste enthielt prüfungsformatspezifische Detailkriterien, die als erwartete Antworten aufgelistet sind (Beispiel):

- *RTA*: Differenzierung zwischen Luft- und Knochenleitung, Frequenzdarstellung, Farbkodierung (rechts/links), Ergänzung um Weber- und Rinne-Test.
- *RMM*: seitengetrennte Testung, Abkleben eines Nasenlochs, Durchführung nach abschwellenden Nasentropfen, Lesart der Kurve bei 150 Pa, Interpretation des Abschwelleffekts.

Exemplarisch ein möglicher Dialog einer Prüfungssituation für die RTA:

### Prüfer:in:

„Was ist auf der Diagnostik dargestellt?“

### Student:in:

„Dargestellt ist der Kurvenverlauf der Hörschwellen über verschiedene Frequenzen. Rot ist die Messung des rechten Ohrs, blau die des linken Ohrs. Die Luftleitung ist als durchgezogene Linie dargestellt, die Knochenleitung als unterbrochene Linie.“

Anhand der Checkliste wird die Antwort auf Vollständigkeit geprüft (5 von 6 möglichen Punkten wurden erreicht):

- ☑ Rot: rechtes Ohr
- ☑ Blau: linkes Ohr
- ☑ Luftleitung: durchgezogene Linie
- ☑ Knochenleitung: unterbrochene Linie
- ☑ Kurvenverlauf über Frequenzen
- ☐ Oben kleine Tabelle: Weber und Rinne

Die Prüfungen wurden im 1:1-Setting durch eine:n von insgesamt 8 ärztlichen Prüfer:innen durchgeführt. Aus einem zuvor festgelegten Pool unterschiedlicher Befunde wurden dabei 2 verschiedene Themen ausgewählt und jeweils 2–3 min geprüft, sodass sich eine Gesamtdauer von 4–6 min pro Prüfung ergab. Die maximal erreichbare Gesamtpunktzahl betrug 27 Punkte.

Für die Auswertung wurden *n* = 407 SMP berücksichtigt (RTA: *n* = 217; RMM: *n* = 190). Die statistische Auswertung erfolgte mit IBM SPSS Statistics (Version 30, IBM Corp., Armonk, NY, USA). Neben einer deskriptiven Analyse wurden Reliabilitäts- und Trennschärfeanalysen (Cronbach‑α, korrigierte Item-Skalen-Korrelationen) durchgeführt. Zur Überprüfung der Konstruktvalidität erfolgte eine explorative Faktorenanalyse (Hauptkomponentenanalyse) getrennt für RTA und RMM. Die Faktorextraktion erfolgte nach dem Kaiser-Kriterium (Eigenwert > 1), die Eignung der Daten wurde mittels Kaiser-Meyer-Olkin-Maß (KMO) und Bartlett-Test auf Sphärizität überprüft. Schließlich wurden mittels einfaktorieller ANOVA („analysis of variance“) mögliche Prüfer:inneneffekte auf die vergebenen Gesamtpunktzahlen analysiert. Die grafische Darstellung der Mittelwerte pro Prüfer:in und Prüfungsformat diente der Visualisierung von Streuung und potenziellen Ausreißern.

## Ergebnisse

### Stichprobe und Prüfungsdurchführung

Im Rahmen des curricularen Blockpraktikums (10. Fachsemester) wurden SMP zu RTA und RMM erhoben. Dabei führten 8 ärztliche Prüfende die Prüfungen im 1:1-Setting durch. Die maximal erreichbare Gesamtpunktzahl betrug 27 Punkte (5 Kategorien; Abzüge für Hilfestellung).

Für die Analyse der Prüfendeneffekte wurden ausschließlich die Prüfungen der Kohorte 2023 berücksichtigt (andere Prüfendengruppe 2024). Prüfende mit < 3 Prüfungen wurden ausgeschlossen. Die Fallzahlen je Prüfendem (gesamt/RTA/RMM) betrugen: P1: 40/9/5; P2: 44/9/9; P3: 94/22/20; P4: 74/17/17; P5: 148/27/34; P6: 22/7/5; P7: 19/5/3; P8: 22/5/5.

Die Auswertung der beiden Kohorten (Jahrgänge 2023, *n* = 209, und 2024, *n* = 198) zeigte keine signifikanten Unterschiede in den Gesamtpunktzahlen der SMP. Für die RTA betrugen die Mittelwerte 25,18 ± 1,76 Punkte in Kohorte 1 (*n* = 101) und 25,16 ± 1,95 Punkte in Kohorte 2 (*n* = 116; *p* = 0,90). Für die RMM lagen die Mittelwerte bei 24,84 ± 1,94 Punkten in Kohorte 1 (*n* = 98) und 25,02 ± 2,04 Punkten in Kohorte 2 (*n* = 92; *p* = 0,49). Da sich die Ergebnisse nicht signifikant zwischen den Kohorten unterschieden, wurden in Tab. 2 die Gesamtmittelwerte über beide Kohorten hinweg angegeben (RTA: 25,20 ± 1,94; RMM: 24,84 ± 1,94; Maximalpunktzahl: 27).

**Tab. 2:** Durchschnittlich erreichte Punktzahlen (±Standardabweichung, SD) in den einzelnen Bewertungskategorien sowie der Gesamtpunktzahl bei der strukturierten mündlichen Prüfung (SMP) für Reintonaudiometrie (RTA, *n* = 217) und Rhinomanometrie (RMM, *n* = 190).

Kategorie	Maximale Punktzahl	RTA (*n* = 217)	RMM (*n* = 190)
1. Benennung der Untersuchung	1	0,98 ± 0,12	1,00 ± 0,00
2. Durchführung der Untersuchung	6	5,95 ± 0,23	5,81 ± 0,52
3. Beschreibung des Befundes	6	5,76 ± 0,62	5,97 ± 0,18
4. Interpretation des Befundes	6	5,85 ± 0,61	5,56 ± 1,05
5. Nennung von Erkrankungen	4	3,84 ± 0,55	3,90 ± 0,43
*Gesamtpunktzahl*	*27*	*25,20* *±* *1,94*	*24,84* *±* *1,94*

### Prüfendeneffekte

Für die Analyse der Prüfer:innenunterschiede wurden ausschließlich die Prüfungen der Kohorte 2023 berücksichtigt; im Jahr 2024 prüfte eine andere Prüfer:innengruppe. Die einfaktorielle ANOVA über beide Fallbeispiele (RTA + RMM) zeigte Unterschiede zwischen Prüfenden in den Gesamtpunktzahlen (*F* = 5,033; *p* < 0,001). Getrennt nach Befunden ergaben sich keine Unterschiede für RMM (*F* = 1,905; *p* = 0,078), jedoch Unterschiede für RTA (*F* = 3,789; *p* = 0,001). Im Tukey-Post-hoc-Test zeigte sich ein Unterschied zwischen P5 und P1 (*p* = 0,0287); zwischen den übrigen Prüfenden (P2, P3, P4, P6, P7, P8) ergaben sich keine signifikanten Unterschiede.

**Abb. 1:**
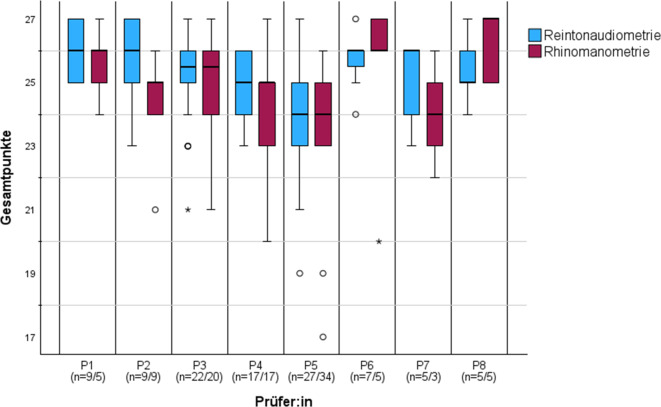
Boxplots der Gesamtpunktzahlen bei der strukturierten mündlichen Prüfung (SMP) für Reintonaudiometrie (*blau*) und Rhinomanometrie (*rot*) nach Prüfer:in (1–8). *Box* = Interquartilsabstand (IQR), *Linie* = Median, Whisker bis 1,5×IQR; ◦ Ausreißer (> 1,5×IQR), *Asterisk* extreme Ausreißer (> 3×IQR). Fallzahlen je Prüfer:in (P1–8) für Reintonaudiometrie (RTA) und Rhinomanometrie (RMM)

Die Abb. 1 zeigt Boxplots der Gesamtpunktzahlen je Prüfendem und Befund (RTA blau, RMM rot). Dargestellt sind Median (horizontale Linie), Interquartilsabstand (IQR; Box) und Whisker bis 1,5×IQR; Ausreißer > 1,5×IQR sind als Kreise (°), > 3×IQR als Sterne (*) markiert. Fallzahlen je Prüfendem sind in der x‑Achsen-Beschriftung angegeben.

### Psychometrische Kennwerte

Die interne Konsistenz lag bei Cronbach-α = 0,54 (RTA) bzw. 0,55 (RMM). Die korrigierten Item-Skalen-Korrelationen lagen überwiegend im niedrigen bis mittleren Bereich (r = 0,22–0,54). In einer explorativen Faktorenanalyse (Hauptkomponentenanalyse) war der Bartlett-Test für beide Befunde signifikant (RTA: χ^2^ = 89,997; *p* < 0,001; RMM: χ^2^ = 36,751; *p* < 0,001), die KMO-Werte betrugen 0,49 (RTA) bzw. 0,50 (RMM).

Für beide Beispiele wurden 2 Faktoren (Eigenwert > 1) extrahiert, die 55,8 % (RTA) bzw. 63,6 % (RMM) der Varianz erklärten. In beiden Prüfungsformaten luden die Kategorien *Benennung, Durchführung* und *Beschreibung* primär auf Faktor 1, während *Interpretation* und *Differenzialdiagnose* auf Faktor 2 luden.

## Diskussion

Unsere Ergebnisse verdeutlichen, dass die SMP als Prüfungsformat in der HNO-Lehre des Medizinstudiums, am Beispiel der RMM und RTA, zuverlässig einsetzbar ist. Die insgesamt hohen mittleren Gesamtpunktzahlen sprechen für eine gute Beherrschung der Inhalte durch die Studierenden. Gleichzeitig verdeutlichen die beobachteten Prüfer:innenunterschiede, insbesondere bei der RTA, die Notwendigkeit konsequenter Standardisierung und regelmäßiger Prüfer:innenschulung, um eine möglichst hohe Objektivität sicherzustellen.

Die SMP nimmt im Kompetenzmodell des NKLM eine besondere Stellung ein. Während schriftliche Prüfungen primär Kompetenzstufe 1 (Faktenwissen) erfassen und praktische Prüfungsformate wie „objective structured clinical evaluation“ (OSCE) auf Stufe 3 (Handlungskompetenz) abzielen, adressiert die SMP gezielt Kompetenzstufe 2 (Handlungs- und Begründungswissen). Sie ermöglicht es, Sachverhalte zu erklären, in klinisch-wissenschaftliche Kontexte einzuordnen und Befunde datenbasiert zu bewerten – Bereiche, die in klassischen Prüfungsformaten bislang kaum abgebildet werden. Damit schließt die SMP die curriculare Lücke zwischen Wissensreproduktion und praktischer Handlungskompetenz und trägt zur Vergleichbarkeit der Ausbildungsqualität sowie zur Vorbereitung auf die ärztliche Weiterbildung bei [1, 2].

### Prüfendeneffekte und -kalibrierung.

In der Analyse der Gesamtpunktzahlen zeigten sich Bewertungsunterschiede zwischen Prüfenden, insbesondere im Format RTA. Eine formale Prüfer:innenkalibrierung (z. B. Ankerfälle, gemeinsame Bewertungsübungen, verhaltensverankerte Deskriptoren) fand in dieser Erhebungsrunde nicht statt. Auffällig ist die deutlich höhere Anzahl an durchgeführten Prüfungen von Prüfer:in 5 (*n* = 148 gesamt) im Vergleich zu den übrigen Prüfenden (40/44/94/74/22/19/22), was auf einen Erfahrungsvorsprung hindeutet. Dies deckt sich mit früheren Arbeiten, die interindividuelle Bewertungsunterschiede bei mündlichen Prüfungen beschreiben [12]. Mit den vorliegenden Daten lässt sich jedoch nicht klären, ob das beobachtete Muster eher Strenge oder Großzügigkeit („leniency“/„severity“) widerspiegelt. Mögliche Ursachen sind unterschiedliche Erwartungshorizonte oder subjektive Maßstäbe bei der Interpretation. Für die RMM ergaben sich hingegen keine signifikanten Unterschiede, was auf eine homogenere Bewertungspraxis oder eine stärker standardisierte Durchführung hindeutet. Für künftige Durchgänge empfiehlt sich daher eine systematische Kalibrierung (gemeinsame Ankerfälle, Kriterienraster mit Verhaltensankern) [9] sowie die Modellierung von Rater-Effekten (z. B. Many-Facet-Ansätze), um individuelle Bewertungsneigungen empirisch zu quantifizieren.

### Ceiling und Differenzierung.

Die hohen Mittelwerte bei gleichzeitig geringer Streuung deuten auf eine begrenzte Differenzierungsbreite in Teilen der Skala hin. Dies muss nicht zwingend für übermäßige Großzügigkeit sprechen, kann aber auf inhaltlich zu leichte Fälle, eine breite Beherrschung der Inhalte oder auf Scoring-Spielräume in einzelnen Kategorien verweisen. Um die Trennschärfe zu erhöhen, sollte die nächste Kohorte gezielt schwierigere Fallvarianten, präzisere Bewertungsanker und ggf. gewichtete Teilpunkte vorsehen [12, 16].

### Einordnung gegenüber alternativen Formaten.

Als ergänzende Formate der Kompetenzstufe 2 bieten sich insbesondere der Skript-Konkordanz-Test (SKT) und das Key-Features-Examen (KFE) an. Der SKT fokussiert klinisches Schlussfolgern unter Unsicherheit und vergleicht Antworten mit einem Expert:innenreferenzschlüssel; das KFE prüft entscheidungsrelevante Schlüsselschritte in klinischen Fällen mit strikt definiertem Scoring. Während die SMP zusätzlich kommunikative Strukturierung, Begründungen „am Fall“ und die Interaktion abbildet, sind SKT und KFE stärker standardisiert und damit weniger anfällig für Rater-Effekte, erfordern aber aufwendige Item‑/Panel-Prozesse. Eine triangulative Kombination (z. B. SMP als mündliches Format plus KFE- oder SKT-Module für Entscheidungs‑/Unsicherheitsmomente) kann die Evidenz für Kompetenzstufe 2 inhaltlich und methodisch verbreitern.

### SMP als formatives Instrument.

Neben der summativen Leistungsbewertung bietet die SMP auch Potenzial als formatives Instrument. Unsere Ergebnisse zeigen, dass individuelle Leistungsprofile sichtbar werden, etwa bei der Befundinterpretation oder der Benennung von Differenzialdiagnosen. Damit kann das Format gezielt für Rückmeldungen an Studierende genutzt werden [11, 17, 18].

### Limitationen.

Limitationen bestehen v. a. in der monozentrischen Anlage der Studie und den unterschiedlichen Prüfungszahlen pro Prüfer:in, die trotz Gewichtung die Aussagekraft einschränken können. Zudem wurden keine Videoaufzeichnungen angefertigt, sodass keine unabhängige Nachbewertung möglich war. Daneben erfolgte die Prüfungsentwicklung ohne Delphi-Verfahren oder formalisiertes Expertenreview; die Validitätsevidenz ist daher explorativ. Für künftige Kohorten ist eine systematische Validierung der SMP erforderlich**.** Dazu zählen eine nachvollziehbare Begründung der beabsichtigten Nutzung und Interpretation der Ergebnisse, klare und einheitliche Bewertungsmaßstäbe mit Prüfer:innenkalibrierung, die Einbindung von Fachexpert:innen zur Überprüfung der Inhalte sowie eine kurze Pilotierung und transparentes Berichten der Gütekriterien.

Für die Lehre ergeben sich konkrete Implikationen: Regelmäßige Prüfer:innenschulungen, die gemeinsame Durchsicht von Beispielprüfungen sowie digitale Bewertungsunterstützung mit hinterlegten Ankerbeispielen könnten die Reliabilität weiter verbessern.

## Ausblick

Zusammenfassend bestätigen unsere Ergebnisse den hohen didaktischen Wert der SMP zur Abbildung von Handlungs- und Begründungswissen im Medizinstudium bei gleichzeitigem Entwicklungsbedarf in Kalibrierung, Validierung und Differenzierung. Durch die gezielte Prüfung von Kompetenzstufe 2 schließt sie die Lücke zwischen reiner Wissensabfrage und praktischer Handlungskompetenz. Setzt man in zukünftigen Runden auf strukturierte Prüfer:innenkalibrierung, erweiterte Validitätsevidenz und eine breitere Aufgabenstreuung, kann das Format einen substanziellen Beitrag zur kompetenzorientierten Leistungsbewertung leisten und durch die Kombination mit SKT/KFE-Elementen weiter an Aussagekraft gewinnen.

## Fazit für die Praxis

- Klassische unstrukturierte mündliche Prüfungen weisen Defizite in Reliabilität und Objektivität auf.
- Die strukturierte mündliche Prüfung (SMP) schließt die Lücke zwischen reiner Wissensabfrage (Kompetenzstufe 1) und praktischer Handlungskompetenz (ab Stufe 3).
- Durch Blueprinting, standardisierte Fallvignetten und Checklisten können Handlungs- und Begründungswissen (Kompetenzstufe 2) valide erfasst werden.
- Prüfer:innenunterschiede unterstreichen die Bedeutung regelmäßiger Schulungen, von Ankerbeispielen und digitaler Bewertungsunterstützung.
- Die SMP eignet sich sowohl für summative Leistungsbewertung als auch für formative Rückmeldung und kann in Lehre und Facharztprüfung einen wertvollen Beitrag leisten.

## Data Availability

Die erhobenen Datensätze können auf begründete Anfrage in anonymisierter Form beim korrespondierenden Autor angefordert werden.
